# Single-photon emission computed tomography of spontaneous liver metastasis from orthotopically implanted human colon cancer cell line stably expressing human sodium/iodide symporter reporter gene

**DOI:** 10.1186/2191-219X-2-46

**Published:** 2012-09-07

**Authors:** Masayuki Inubushi, Yong-Nan Jin, Chika Murai, Hironobu Hata, Yoshimasa Kitagawa, Tsuneo Saga

**Affiliations:** 1 Molecular Imaging Center, National Institute of Radiological Sciences, 4-9-1 Anagawa, Inage-ku, Chiba, 263-8555, Japan; 2 Oral Diagnosis and Oral Medicine, Graduate School of Dental Medicine, Hokkaido University, Kita 13 Nishi 7, Kita-ku, Sapporo, 060-8586, Japan

**Keywords:** Molecular imaging, Animal model, Spontaneous liver metastasis, Colorectal neoplasms, Radionuclide reporter gene

## Abstract

**Background:**

We aimed to develop a mouse spontaneous liver metastasis model from an orthotopically implanted human colon cancer cell line stably expressing a human sodium/iodide symporter (NIS) reporter gene, which can be imaged with single-photon emission computed tomography (SPECT) using ^99m^TcO_4_^−^.

**Methods:**

A recombinant plasmid containing a constitutively driven NIS gene (pcDNA3-NIS) was transfected into the human colon cancer cell line HCT116, and stable cell lines were established. The stable cells were subcutaneously injected into the nude mice. When the diameter reached 10 mm, the xenografts were excised, cut into small fragments, and orthotopically implanted into the cecal walls of another nude mice. ^99m^TcO_4_^−^ SPECT/CT imaging was initiated 8 weeks later and repeated every 1 to 2 weeks.

**Results:**

The production and function of NIS protein was confirmed *in vitro* by Western blotting and ^99m^TcO_4_^−^ uptake assay. On SPECT/CT imaging, focal ^99m^TcO_4_^−^ uptake was detected in the liver. Necropsy revealed local growth of the orthotopic colon xenografts with extensive invasion, microscopic serosal metastasis, and metastatic foci in the corresponding hepatic regions showing focal ^99m^TcO_4_^−^ uptake. Immunohistochemistry revealed high levels of NIS expression in cells forming liver tumor, indicating that the liver tumor cells originated from the orthotopic colon xenografts.

**Conclusions:**

The present proof-of-concept study provided a rationale for employing a radionuclide reporter gene for the specific visualization of spontaneous liver metastasis in living mice. This unique animal model of clinically relevant and externally detectable liver metastasis will be a powerful tool for investigating tumor biology and developing novel therapies for cancer metastasis.

## Background

Research using animal models that represent all processes of metastasis formation is essential. However, the currently used rodent tumor models, such as the subcutaneous (ectopic) tumor graft models in nude mice, do not sufficiently represent clinical cancer, particularly with regard to metastasis formation, since the subcutaneous micro-environment for visceral tumors is considerably different from their original environment [1,2]. Experimental liver metastasis can be induced by intrasplenic [3] or direct intraportal [4] injection of cancer cells in the nude mice; however, a major disadvantage of this procedure is that the early stages of metastasis formation, such as local invasion at the site of the primary tumor and gaining access to lymphatics or blood vessels, are bypassed [5]. Hence, to reproduce clinically relevant spontaneous tumor metastasis, tumor cells should be implanted into the correct anatomical (orthotopic) sites of immunodeficient mice. However, this animal model has a drawback - poor predictability of spontaneous metastasis formation from the orthotopic primary tumor. These tumor cells have sometimes been labeled with fluorescent [6,7] or bioluminescent [8,9] reporter genes to enable specific detection, but the poor penetration of light within the mammalian tissue limits the use of fluorescent and bioluminescent imaging to superficial lesions. Detecting a metastatic tumor in the mouse liver at any level deeper than 1 cm would be extremely difficult using fluorescent and bioluminescent imaging because approximately 90% of optical signal flux is lost per centimeter of mammalian tissue [10]. Moreover, cells expressing fluorescent proteins remain detectable even after the cells die, which may prevent from assessing therapeutic response of the metastatic tumor.

To overcome this drawback, labeling tumor cells with radionuclide reporter genes appears to be ideal since their reporter products can be quantitatively and three-dimensionally imaged even deep within the body using corresponding radiolabeled reporter probes and positron emission tomography or single-photon emission computed tomography (SPECT). Among several radionuclide reporter genes proposed to date, the human sodium/iodide symporter (NIS) reporter gene appears to be the most promising [11]. Nevertheless, there has been no report to date on the application of the NIS reporter gene for such metastatic cell tracking, and there is no guarantee that it would be effective. It is unclear how *in vivo* tumor growth can be delayed by the NIS gene transfer despite the additional constitutive production of reporter products, which often leads to a slower rate of tumor growth than in the wild type. It is also unclear how much metastatic potential is maintained in implanted tumor cells transferred with the NIS gene because intracellular levels of sodium ions as well as iodide may be increased in such cells, and this may affect the complex steps involved in the formation of metastases. In the present study, we therefore aimed to develop a mouse spontaneous liver metastasis model from an orthotopically implanted human colon cancer cell line stably expressing NIS reporter gene and to demonstrate the feasibility of SPECT reporter gene imaging of the metastatic lesions using ^99m^TcO_4_^–^.

## Methods

### Vector construction, cell culture, stable gene transfer, and selection

A human NIS cDNA [GenBank:NM000453] was synthesized by polymerase chain reaction and cloned into the *Eco*RI site of the multicloning site of pcDNA3 vector (Invitrogen, Carlsbad, CA, USA) (pcDNA3-NIS) [12]. The human colon cancer cell line HCT116 (American Type Culture Collection, Manassas, VA, USA) was used in this study since it has been reported to have the highest metastatic potential among the ten human gastrointestinal cancer cell lines that have been studied [3]. The cells were cultured in DMEM/F-12 (Invitrogen) supplemented with 10% fetal bovine serum, 100-IU/ml penicillin, and 50-μg/ml streptomycin. For stable gene transfer, HCT116 cells were transfected with pcDNA3-NIS by the calcium phosphate coprecipitation method (pH 7.12) as previously described in [13]. For the selection, the cells were cultured in the presence of 1,000-μg/ml G418 (Invitrogen). Approximately 80 drug-resistant colonies were isolated and subcloned; three of these were expanded for further analyses (cell lines A, B, and C), where a non-transfected HCT116 cell line served as the negative control (cell line N).

### Western blotting

NIS protein expression in both stable and negative control cell lines was examined by Western blotting as previously described in [14]. Briefly, the membrane protein fraction extracted from the cell lysates was assayed using a mouse monoclonal anti-human NIS antibody (clone FP5A; Lab Vision, Fremont, CA, USA) at 1:2,000 dilution. When NIS protein expression was detected by Western blot, NIS protein levels were quantitatively determined from the optical density measurements and normalized to α-tubulin protein levels.

### *In vitro*^99m^TcO_4_^–^ uptake assay

The function of NIS protein in both stable and negative control cell lines was confirmed by ^99m^TcO_4_^−^ uptake assay as previously described in [15]. Briefly, 24 h after seeding at 5 × 10^4^ cells/well on 24-well plates, the cells were incubated with a medium containing 3.7 kBq/well of ^99m^TcO_4_^−^ for 30 min, washed gently and quickly twice with ice-cold HBSS after removing the medium, and then lysed with 0.5 ml of 0.1 N NaOH. *In vitro*^99m^TcO_4_^−^ uptake levels were measured in terms of radioactivity in the cell lysates relative to the total radioactivity added and represented as a percentage. Furthermore, inhibition of ^99m^TcO_4_^−^ uptake was confirmed using 50-μM sodium perchlorate, a competitive inhibitor of NIS. These experiments were performed in quadruplicate and repeated twice.

### Animals

Seven-week-old female nude mice (BALB/c A Jcl-nu/nu; CLEA Japan, Tokyo, Japan) were used in all animal experiments. All animals were humanely treated in accordance with the *Guide for the Care and Use of Laboratory Animals* formulated by the US National Research Council [16]. Mice were fed a commercial diet MB-1 (Funabashi farm, Funabashi, Japan) that was not supplemented with iodine. The study was conducted in accordance with our institutional *Guide for the Care and Use of Laboratory Animals* (publication no. 7–35, revised in 2011).

### Small animal SPECT/CT imaging protocol

The small-animal SPECT/CT system FX (Gamma Medica-Ideas, Northridge, CA, USA) was used along with the pinhole collimator PH10 (Gamma Medica-Ideas). Mice were anesthetized with 1% to 2% isoflurane and heated by a lamp to maintain body temperature during the entire scanning period. CT images were obtained for anatomical orientation immediately before SPECT imaging. For SPECT imaging, 30 min after intravenous injection of 111 MBq of ^99m^TcO_4_^−^ (Fujifilm RI Pharma, Tokyo, Japan), 64 projections were acquired for 1 min per projection at the shortest radius of rotation (30 mm) in a 140 keV ± 10% energy window. SPECT images were reconstructed using ordered subset expectation maximization (5 iterations, 4 subsets) without attenuation correction using the FLEX SPECT software (Gamma Medica-Ideas). The reconstructed images were consistent with 80 × 80 image matrices and had a spatial resolution of 0.8 mm. For quantitative analysis, a spherical region of interest was placed so that a focal ^99m^TcO_4_^−^ uptake to the tumor was entirely covered, and *in vivo*^99m^TcO_4_^−^ uptake levels were estimated as standardized uptake value (SUV) using a cross-calibration factor measured in advance with a cylindrical phantom and assuming a tissue specific gravity of 1 g/ml.

### *In vivo* studies using mouse subcutaneous xenograft model

Three stable and one negative control cell lines were harvested by trypsinization and washed three times with cold serum-free medium. The four types of cell suspension (3 × 10^6^ cells/0.2 ml) were subcutaneously injected in a rotating order in both shoulders and thighs of the 15 mice, and each subcutaneous xenograft was assessed separately.

To evaluate whether NIS transfer affected *in vivo* tumor growth, xenograft growth was assessed by callipering the maximum body surface diameter every 2 to 3 days for up to 2 months. The rate of tumor formation was calculated for each cell line as the number of tumors having diameter ≥8 mm divided by the number of subcutaneous injection sites (*n* = 15). The threshold diameter of the tumor formation was set to 8 mm because the tumor shrinkage and disappearance had been recorded in tumors having diameter <8 mm. The mean time to 8-mm tumor formation was also determined excluding tumors which failed to grow up to 8 mm within 8 weeks.

To confirm whether the functional characteristics of ^99m^TcO_4_^−^ uptake were preserved *in vivo*, SPECT/CT imaging was performed in the subcutaneous xenograft model mice whenever each diameter of the four xenografts reached 8 mm.

### *In vivo* studies using mouse orthotopic xenograft model

Cells (3 × 10^6^) from one of the stable cell lines and the negative control cell line were each subcutaneously injected in the thigh of 15 nude mice. When the diameter of the xenografts reached 10 mm, they were excised, cut into small fragments (25 to 30 mg), and orthotopically implanted into the cecal walls of another 15 nude mice for each cell line, as previously described in [17].

During follow-up, the experimental endpoint was defined as the detection of metastasis on SPECT/CT imaging and the humane endpoints as the heavy primary tumor burden, paraneoplastic conditions (e.g., weight loss >20%, respiratory difficulties), and pain/discomfort because of tissue destruction or distension. Mice were euthanized at the earliest endpoint.

SPECT/CT imaging was initiated 8 weeks after orthotopic implantation since some mice appeared to have reached the humane endpoints around this time point. Imaging was repeated every 1 to 2 weeks for as long as possible.

### Pathological necropsy

Complete necropsy was performed after euthanasia in all mice in the orthotopic xenograft model. The cervical, thoracic, abdominal, and pelvic organs were extracted en bloc and sectioned in case of the need for microscopic assessment of tumor tissue. The liver was entirely sectioned at 2-mm thickness, and metastatic foci were searched thoroughly by macroscopic inspection and palpation of the sections so that all metastatic foci larger than 1 mm could be detected.

### Immunohistochemistry

Tumor specimens were dissected, fixed in formalin, embedded in paraffin, cut into 4-μm sections, immunostained with primary mouse monoclonal anti-human NIS antibody (clone FP5A; Lab Vision) at 1:100 dilution and secondary anti-mouse immunoglobulin/horseradish peroxidase, and stained brown with 3,3 ^′^-diaminobenzidine HCl according to the manufacturer’s instructions. The nuclei were stained with Mayer’s hematoxylin.

### Statistical analysis

Data are presented as mean ± SD. Two-way repeated measure ANOVA with post hoc Bonferroni test was used to compare mean values among three or four groups. Unpaired Student’s *t*-test was used to compare mean values between two groups. In Chi-square test, a contingency table was used to compare rates or ratios between two to four groups. The Kaplan-Meier method was used to generate survival curves, and the log-rank (Mantel-Cox) test was used to compare the survival curves. All statistical analyses were performed using Prism 5 for Mac OS X (GraphPad Software, La Jolla, CA, USA). *p* values <0.05 were considered statistically significant.

## Results

### Western blotting

**Figure 1:**
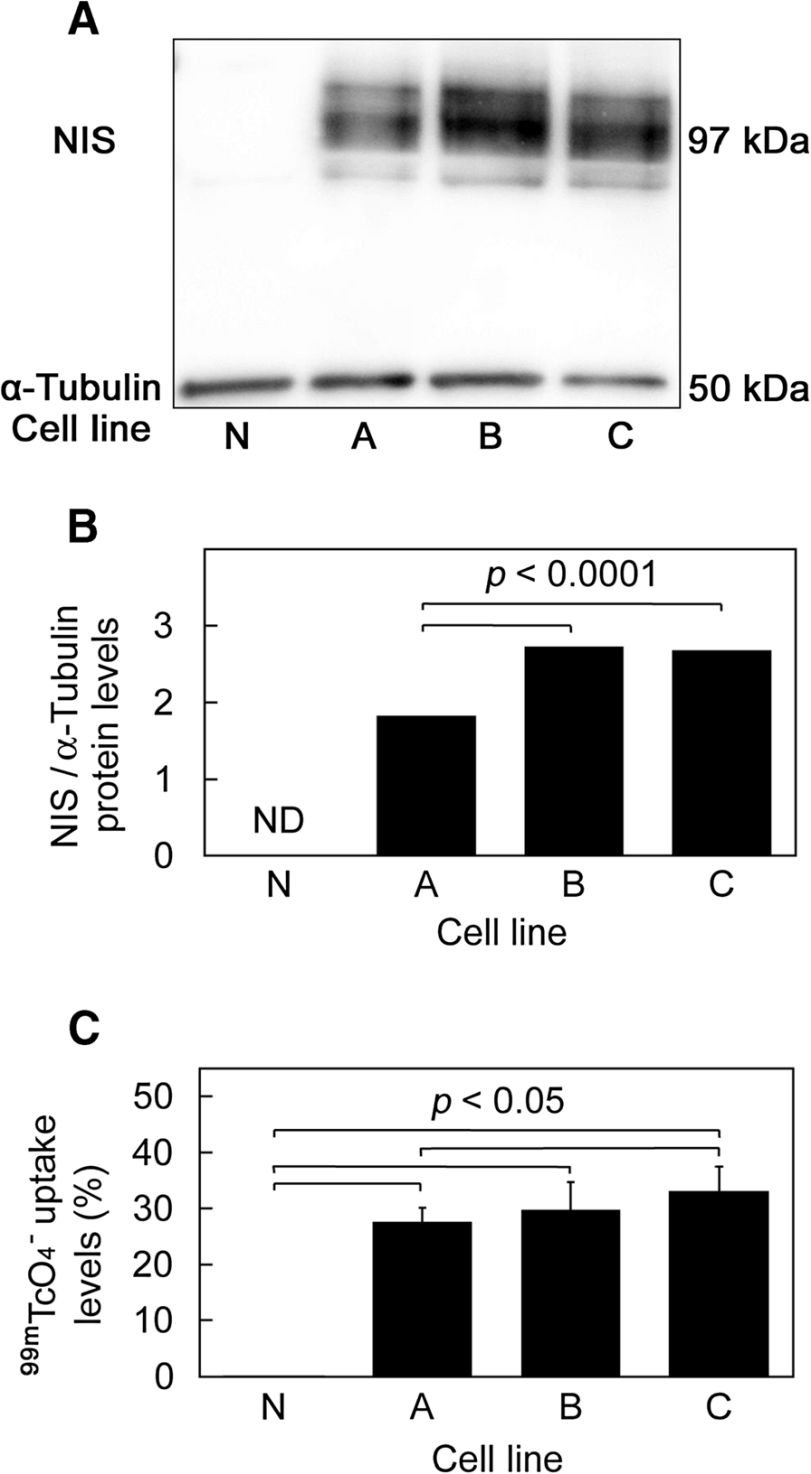
***In vitro *studies. ** (**A**) Western blot of NIS protein, (**B**) NIS protein levels normalized to α-tubulin protein levels, and (**C**) *in vitro*^99m^TcO_4_^−^ uptake assay. Cell lines A, B, and C (black bars) were HCT116 stably transfected with pcDNA3-NIS, and cell line N (white bar) was the untransfected negative control. ND, not detectable.

NIS protein was recognized at approximately 97 kDa on Western blots from the three selected stable cell lines A, B, and C, but not from the negative control cell line N (Figure 1A,B). The wide bands for NIS protein were probably attributable to the preparation method from the membrane fraction, as previously reported in [18]. The ratio of NIS protein levels to α-tubulin protein levels was 1.82 in line A, 2.71 in line B, and 2.67 in line C.

### *In vitro*^99m^TcO_4_^–^ uptake assay

*In vitro*^99m^TcO_4_^–^ uptake assay demonstrated high uptake levels of ^99m^TcO_4_^–^ by all three stable cell lines (A: 27.50 ± 2.58%, B: 29.68 ± 5.07%, C: 33.02 ± 4.52%), but not by the negative control cell line (N: 0.03 ± 0.01%) (Figure 1C). The high ^99m^TcO_4_^–^ uptake levels were almost completely inhibited by sodium perchlorate (A: 0.09 ± 0.03%, B: 0.08 ± 0.04%, C: 0.08 ± 0.05%).

### *In vivo* studies using mouse subcutaneous xenograft model

**Figure 2:**
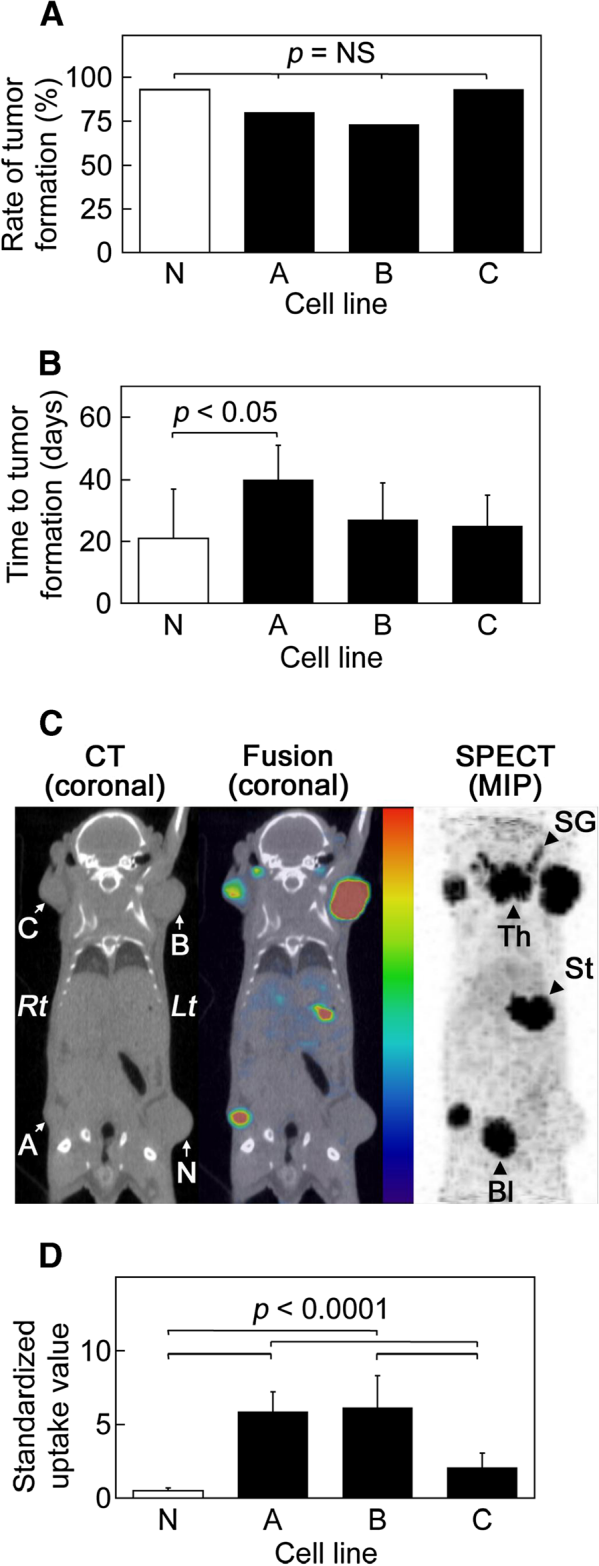
***In vivo *studies using mouse subcutaneous xenograft model. ** (**A**) Rate of tumor formation, (**B**) time to tumor formation, (**C**) SPECT/CT images of a representative mouse. SG, Th, St, and Bl (arrowheads) indicate the salivary glands, thyroid, stomach, and urinary bladder, respectively. Lt, left; MIP, maximum intensity projection; Rt, right. (**D**) ^99m^TcO_4_^–^ uptake levels of subcutaneous xenografts. Cell lines A, B, and C (black bars, arrows) were HCT116 stably transfected with pcDNA3-NIS, and cell line N (white bar, arrow) was the untransfected negative control.

*In vivo* tumor growth was assessed in the subcutaneous xenograft model mice. No significant difference was observed in the rate of tumor formation among the four cell lines (N: 93%, A: 80%, B: 73%, C: 93%) (Figure 2A). The time to formation of tumors ≥8 mm in the three stable cell lines (A: 40 ± 11 days, B: 27 ± 12 days, C: 25 ± 10 days) was slightly greater than that in the negative control cell line (N: 21 ± 16 days). A statistically significant difference was observed only between cell lines A and N (*p* < 0.05, Figure 2B).

SPECT/CT imaging revealed intense ^99m^TcO_4_^–^ uptake by the subcutaneous xenografts generated from cell lines A, B, and C, but not by those generated from cell line N (Figure 2C). The background levels of ^99m^TcO_4_^–^ were consistently low except for the physiological uptake by the salivary glands, thyroid gland, stomach, and urinary bladder. To minimize the effect of tumor size, quantitative uptake analysis was performed for tumors having diameter 8 to 13 mm (*n* = 11 to 14/cell line). ^99m^TcO_4_^–^ uptake levels were significantly higher in the subcutaneous xenografts generated from cell lines A (SUV = 5.8 ± 1.4) and B (6.1 ± 2.2) than in those generated from cell lines C (2.0 ± 1.0) and N (0.5 ± 0.2) (*p* < 0.001, Figure 2D).

Based on the results of these experiments conducted using subcutaneous xenograft model mice, we decided to use cell line B, which showed both rapid tumor growth and high *in vivo*^99m^TcO_4_^–^ uptake levels (11.8-fold of line N), from the three stable cell lines for generating mouse orthotopic xenograft model.

### *In vivo* studies using mouse orthotopic xenograft model

**Figure 3:**
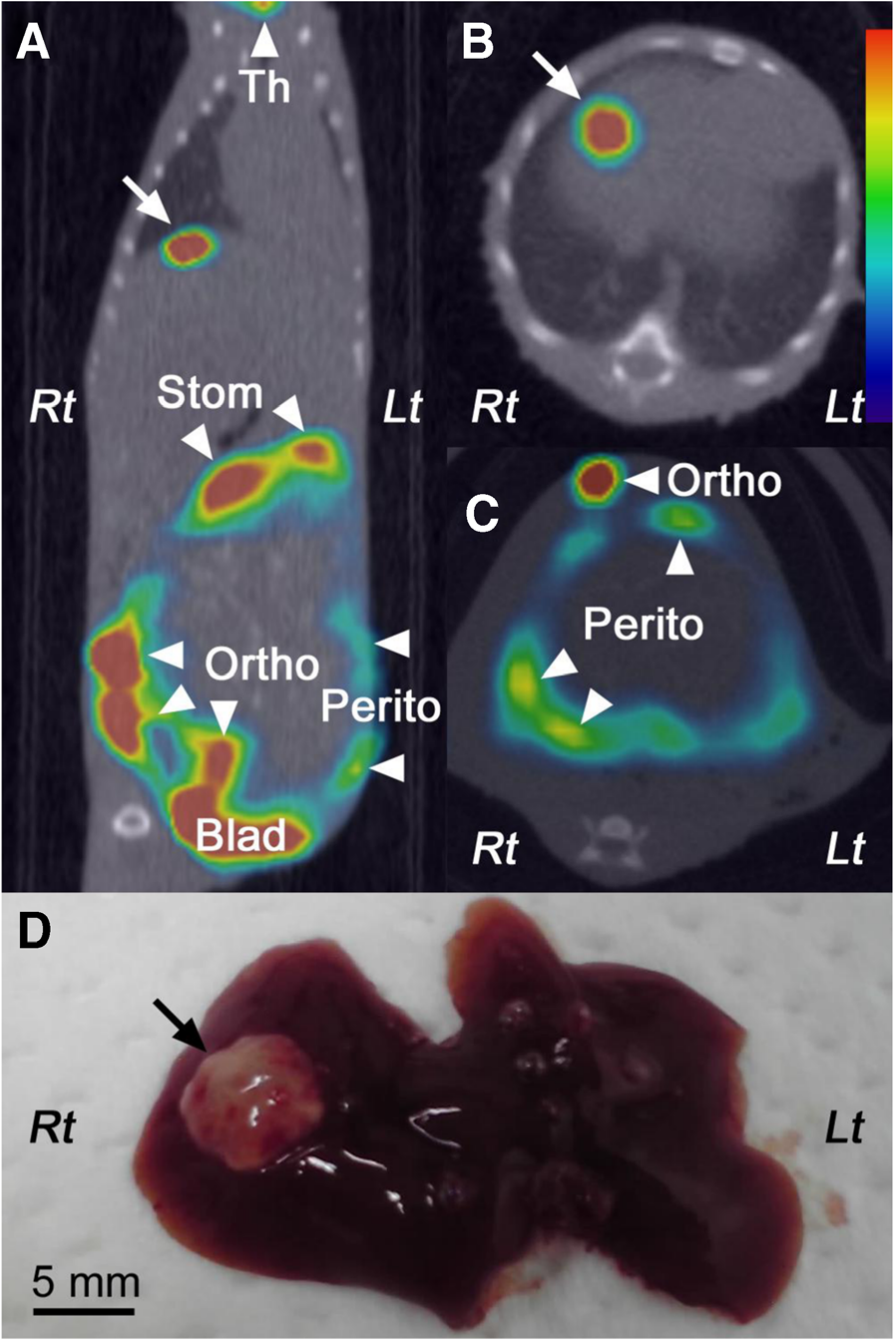
**SPECT/CT fusion images and the liver surface.** (**A**) Transaxial and (**B**),(**C**) coronal sections of SPECT/CT fusion images and (**D**) macroscopic view of the liver surface of a representative mouse orthotopically implanted with a line B tumor fragment. Focal ^99m^TcO_4_^−^ uptake (white arrows; SUV = 5.71) on SPECT/CT images corresponds to tumor cell infiltration (black arrow) in the macroscopic view. Th, Stom, Ortho, Perito, and Blad (arrowheads) indicate the thyroid, stomach, orthotopically implanted primary tumor, peritoneal dissemination, and bladder, respectively. Lt, left; Rt, right.

In all 15 mice implanted with line B tumor fragments, SPECT/CT imaging was performed one to three times per mouse until the experimental or humane endpoint. A focal ^99m^TcO_4_^–^ uptake was found in the right lower abdomen of 12 (80%) of the 15 mice, which corresponded to the orthotopic xenograft. Faint and intermittent ^99m^TcO_4_^–^ uptakes along the abdominal wall were also observed in the same 12 mice, which were speculated to be uptake to the peritoneal dissemination of the cancer cells (Figure 3C). Furthermore, in three (20%) of these 12 mice, focal ^99m^TcO_4_^–^ uptakes were detected in the liver, which were estimated to be liver metastases, although none of them were detected on CT images. The maximum SUV of the focal uptakes in the liver was 5.71, 4.75, and 2.21 (Figure 3A,B). In contrast, the remaining three mice (20%) did not display any abnormal uptake of ^99m^TcO_4_^–^ in the abdomen, which were judged as engrafting failures.

**Figure 4:**
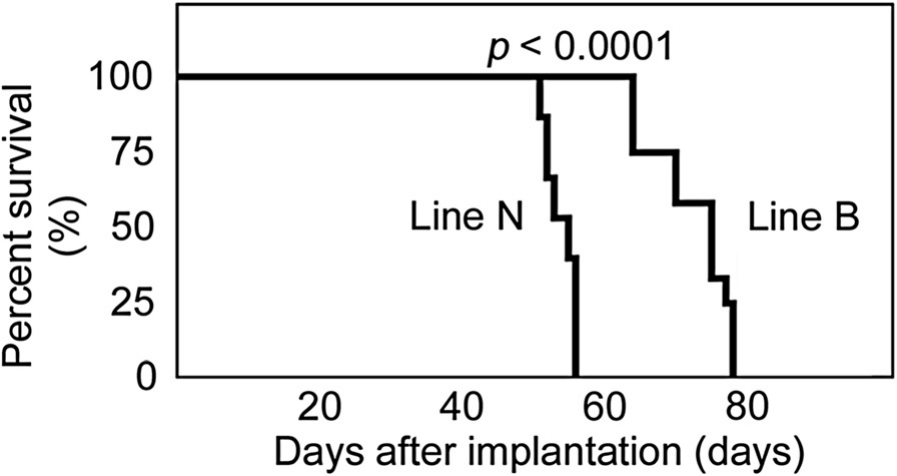
**Survival curves.** Of mice orthotopically implanted with line B tumor fragments (*n* = 12) and those implanted with line N negative control fragments (*n* = 15). Three mice implanted with line B tumor fragments and euthanized because of the absence of physical signs of tumor development were excluded from this analysis. Median survival times were 75 days for line B and 55 days for line N (*p* < 0.0001).

On the other hand, in 12 of the 15 mice implanted with line N negative control tumor fragments, SPECT/CT images were obtained only once, and no abnormal ^99m^TcO_4_^–^ uptake was observed. The remaining three negative control mice reached a humane endpoint and were thus euthanized before SPECT/CT imaging. The mice with cell line N tumor fragments reached humane endpoints faster than those with line B tumor fragments, and all the mice were euthanized at 9 weeks after implantation (Figure 4).

### Pathological necropsy

In the three mice implanted with line B tumor fragments and euthanized because of the absence of physical signs of tumor development, pathological necropsy demonstrated that the fragments had not been taken up at the orthotopic sites. In the remaining 27 mice, the orthotopically implanted tumors resulted in local growth with extensive invasion as well as microscopic serosal metastasis. The severe peritoneal adhesion hampered abdominal tissue collection for biodistribution of ^99m^TcO_4_^−^ after *in vivo* systemic administration.

Many white dots (approximately 100 μm) indicating small metastases were macroscopically observed on the peritoneum and the surface of the liver, but they were below the detectable size by SPECT/CT imaging. We believe that all metastatic foci larger than 1 mm (approximately 1 to 2 mm) were detected by the thorough pathological investigation of the liver metastases. Three foci found in mice with line B fragments (approximately 2 to 5 mm) corresponded to the focal ^99m^TcO_4_^−^ uptake on SPECT/CT images (Figure 3D). Other four metastatic foci found by pathological necropsy were in the mice with negative control fragments. No apparent metastases were detected at other sites such as the lungs and regional lymph nodes.

### Immunohistochemistry

**Figure 5:**
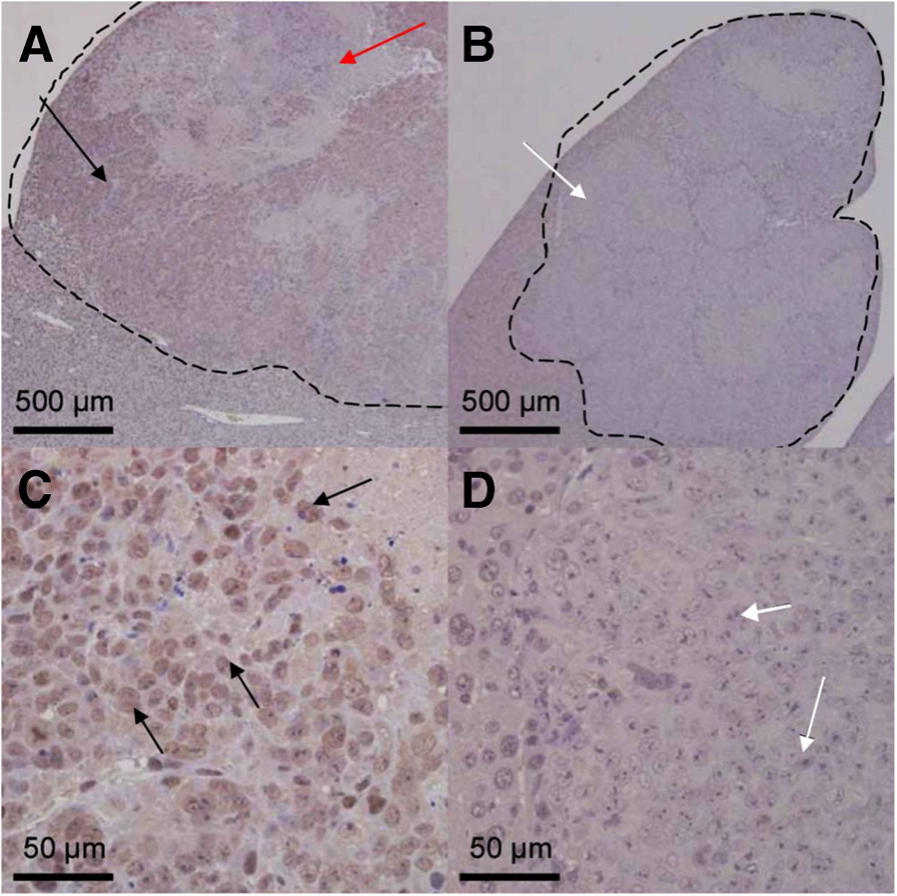
**Immunohistochemistry.** Demonstrating NIS in the liver tumor tissue of representative mice implanted with a line B tumor fragment (**A** and **C**) and a line N negative control fragment (**B** and **D**). Areas bordered by dotted lines indicate the liver tumors. High levels of NIS expression were observed in cells (black arrows) forming the liver tumor except for intratumoral necrotic regions (red arrow) of the mice with line B tumor fragments, but no NIS expression was observed in the tumor cells (white arrows) in the liver of mice implanted with line N fragments.

Immunohistochemical staining of the liver tumor tissue from the mice orthotopically implanted with line B tumor fragments revealed high levels of NIS expression in cells forming the liver tumor except the intratumoral necrotic regions (Figure 5). Since NIS proteins were constitutively produced in the cytoplasm and then moved to the membrane, the entire cytoplasm as well as the membrane was stained brown, but they could not be discriminated. In contrast, no NIS expression was observed in the liver tumor cells of mice implanted with negative control tumor fragments. These findings confirm that the liver tumor cells originated from the orthotopic colon xenografts.

## Discussion

In the *in vitro* experiments, we established three stable cell lines from three single cells transduced with NIS reporter gene and confirmed the production and function of NIS protein by Western blotting and ^99m^TcO_4_^–^ uptake assay. In the following *in vivo* studies using the mouse subcutaneous xenograft model, we found significant growth delay in line A compared with line N. It is a common phenomenon that compared with nontransferred cells, growth delay is observed in cells stably transduced with an exogenous gene driven by a constitutive promoter, probably because gene-transferred cells shoulder the burden of continuously producing its additional protein. In addition, *in vivo*^99m^TcO_4_^–^ uptake levels of xenografts were not consistent with their *in vitro*^99m^TcO_4_^–^ uptake levels because various host factors such as vascularity and local immunity might have affected ^99m^TcO_4_^–^ uptake *in vivo*. Our results indicated that compared with the original cell line, the growth delay was minimal and not statistically significant in lines B and C and that *in vivo*^99m^TcO_4_^−^ uptake to xenografts was preserved in lines A and B. Finally, in the *in vivo* studies using the mouse orthotopic xenograft model with line B, we, for the first time, succeeded in tomographic visualization and quantitative assessment of the clinically relevant spontaneous liver metastases from the colon cancer in living mice. The metastatic potential to the liver from the orthotopic tumor made with line B (3/12 mice = 25%) was comparable to that made with the control line N (4/15 mice = 26.7%). The time to metastatic formation and death was prolonged in the mice implanted with line B compared with those with line N. This dissociation might be related to the fact that the time to tumor formation of cell line B tended to be longer than that of cell line N. However, it will not be a critical problem because the median survival times were within the reported range of 1 to 4 months in both groups [19] and also because in future applications line B mice with intervention (e.g., novel therapy) will be compared with line B mice without intervention instead of line N mice.

The most common way to perform these experiments is to employ a lentiviral construct under conditions of nearly 100% transduction efficiency or to use a bicistronic construct encoding both a radionuclide reporter gene and a fluorescent reporter gene such as GFP to select a transduced population of cells using a fluorescence-activated cell sorter. However, neither of the transduction efficiency or the selection specificity is expected to be completely 100%. Accordingly, if one uses such a polyclonal population of NIS-transduced cells in a similar study, it would be speculated that a small population of cancer cells not transduced with a reporter gene will gradually increase in the primary tumor and may dominantly form metastases, since non-transduced cells are generally faster in tumor growth and metastatic formation compared with transduced cells as shown in our study as well. Also, it would be feared that individual variations become larger among animals, since tumor growth and *in vivo*^99m^TcO_4_^−^ uptake levels vary among clones of NIS-transduced cells as demonstrated in our study. In contrast, we used a clone from a single cell transduced with the reporter gene to assure that all cells were transduced with NIS reporter gene and had homogeneous characteristics, which we believe was the key advantage in the current study.

In addition, using SPECT, we succeeded in clearly visualizing and quantitatively assessing focal ^99m^TcO_4_^−^ uptake in the liver tumors of mice orthotopically implanted with NIS-expressing colon cancer fragments. We also demonstrated, as the *in vivo* proof of concept, by pathological necropsy and NIS immunohistochemistry that these were spontaneous liver metastases from the orthotopic colon xenografts. Several recent studies have applied spontaneous metastasis models to breast cancer, melanoma, lung cancer, and colon cancer, although not by labeling with reporter genes to test new therapeutic regimens because conventional subcutaneous primary tumor xenograft models are often poorly predictive of the therapeutic effects of new drugs [20]. One previous study using a spontaneous metastasis model for breast cancer reported that orthotopic primary tumors significantly responded to trastuzumab monotherapy, whereas minimal antitumor effect was observed when mice with metastatic disease were treated [21]. In contrast, another study reported that a combination of 5-fluorouracil prodrug and cyclophosphamide showed striking therapeutic effects on advanced spontaneous metastatic disease, but not on the primary orthotopic breast tumors [22]. These discrepancies suggest that anticancer drug activity requires evaluation in an appropriate metastasis model for the development and assessment of new therapeutic regimens. As spontaneous metastasis models reproduce all the events involved in the multistep process of the metastatic cascade, they should also help to improve our understanding of the mechanisms that regulate metastatic spread and growth [20].

In spite of the advantage of using spontaneous metastasis models, certain challenges associated with the setting up of the experiments have been also reported [20]. First, although they argued for the surgical removal of orthotopic primary tumors of lung cancer or melanoma at a considerable size (400 to 500 mm^3^) to prevent the rapidly growing primary tumors from causing end-point termination of the experiment, tumors may grow asynchronously making it difficult to decide when surgery should be performed. Second, spontaneous metastases appear unevenly over the experimental time course, although such complexity may hold an advantage in that individual differences in metastatic patterns are more realistic and reflective of the clinical presentation of metastases. Our approach to tomographically visualize both of the primary and metastatic tumors by stably transferring the NIS reporter gene into tumor cells will add a practical aspect to spontaneous metastasis models with regard to solving these issues.

Finally, the present study still has a potential limitation. We found spontaneous liver metastases developed in only three (25%) of 12 mice with local invasion of the reporter fragments. The metastatic rate was relatively low compared with the level of 47% to 60% as reported in previous studies using HCT116 cell lines [19,23,24]. The major obstacle in our study related to this discrepancy seemed to be difficulty due to the severe peritoneal adhesion in resecting the orthotopically implanted primary colon tumor to interrupt the rapid growth, and in fact, 12 of the 15 mice with the reporter tumor fragments had to be euthanized at the humane endpoints rather than the experimental endpoint. Thus, another cell line with a slower growth rate (e.g., cell line A) might be used preferably for slower progression of primary tumors only if it preserves high metastatic potential. Alternatively, the metastatic potential of the given cancer cells should be reinforced by serial *in vivo* selection to facilitate metastasis formation. A previous report described that 4 cycles of selection yielded their cell lines with a very high metastatic efficiency [25]. We are planning to address this limitation in the future; however, for the time being, this limitation could be offset by performing the experiment with more number of mice per group.

## Conclusion

Despite the need for improvement in the efficiency of metastasis formation, the current proof-of-concept study provided a rationale for applying a radionuclide reporter gene for the specific visualization of spontaneous liver metastasis in living mice. Compared with the previous methods utilizing fluorescent or bioluminescent reporter genes, our approach utilized a fully 3D modality with high quantitative accuracy and high sensitivity particularly deep within the body. This unique animal model of clinically relevant and externally detectable liver metastasis will be a powerful tool for investigating tumor biology and developing novel therapies for human colon cancer metastasis.

## Competing interests

The authors declare that they have no competing interests.

## Authors’ contributions

MI developed the concept, acquired the funds, designed the experiments, carried out the molecular genetic studies, instructed YNJ, CM, and HH, interpreted and analyzed the data, and wrote the paper. YNJ performed Western blotting, *in vitro*^99m^TcO_4_^−^ uptake assay, and *in vivo* studies using the mouse orthotopic xenograft model. CM performed *in vivo* studies using the mouse subcutaneous xenograft model. HH participated in the molecular genetic studies. YK acquired the funds and gave critical advises. TS generally supervised the study. All authors read and approved the final manuscript.
